# Effect of Poling on Multicatalytic Performance of 0.5Ba(Zr_0.2_Ti_0.8_)O_3_-0.5(Ba_0.7_Sr_0.3_)TiO_3_ Ferroelectric Ceramic for Dye Degradation

**DOI:** 10.3390/ma15228217

**Published:** 2022-11-18

**Authors:** Akshay Gaur, Shivam Dubey, Zainab Mufarreh Elqahtani, Samia ben Ahmed, Mohammed Sultan Abdulghaffar Al-Buriahi, Rahul Vaish, Vishal Singh Chauhan

**Affiliations:** 1 School of Mechanical and Materials Engineering, Indian Institute of Technology Mandi Himachal Pradesh, Himachal Pradesh 175005, India; 2 Department of Physics, College of Science, Princess Nourah bint Abdulrahman University, P.O. Box 84428, Riyadh 11671, Saudi Arabia; 3 Department of Chemistry, College of Sciences, King Khalid University, P.O. Box 9004, Abha 62529, Saudi Arabia; 4 Department of Physics, Sakarya University, Sakarya 54050, Turkey

**Keywords:** ferroelectric material, multicatalytic, dye degradation, corona poling, photocatalysis, piezocatalysis

## Abstract

Ferroelectric materials with a spontaneous polarization are proven to be potential multicatalysts in water remediation applications. The composition of 0.5Ba(Zr_0.2_Ti_0.8_)O_3_-0.5(Ba_0.7_Sr_0.3_)TiO_3_ (BST-BZT) was examined for photocatalysis, piezocatalysis, and piezo-photocatalysis processes by degrading an azo dye named methylene blue (MB). Generally, dis-aligned dipoles restrict the catalytic activities due to which the BST-BZT powder sample was poled by the corona poling technique. Coupled piezocatalysis and photocatalysis process, i.e., the piezo-photocatalysis process has shown maximum dye degradation. There was a significant improvement in degradation efficiency by using a poled BST-BZT sample compared to the unpoled sample in all processes, thus the results suggest an extensive scope of poled ferroelectric ceramic powder in the catalysis field.

## 1. Introduction

Photocatalysis is one of the most popular advanced oxidation processes for water-cleaning applications. Titanium oxide (TiO2) is a well-studied photocatalyst with a high stability and excellent photochemical properties [1,2]. However, due to low visible light absorption capacity, TiO2 as a photocatalyst is unable to harness the free availability of visible light. Furthermore, the electron-hole pair recombines causing a surface defect, which reduces the efficiency of energy conversion [3,4,5]. Surface functionalization, heterojunction formation, and tuning defects are some of the strategies that can be used to improve the overall efficiency of the photocatalysis process [6,7]. However, ferroelectric materials with inherent spontaneous polarization are recently being acknowledged as effective photocatalysts in electrochemical processes; SrTiO_3_, PbTiO_3_, BaTiO_3_, and BiFeO_3_ are examples of ferroelectric materials [8,9,10,11]. Ferroelectric materials have shown superior catalytic performances in view of hydrogen generation, dye degradation, and antibacterial properties [12,13,14,15,16]. Moreover, these families of materials have demonstrated multicatalytic processes, which include photocatalysis, piezocatalysis, pyrocatalysis, and a combination of these [17,18,19,20]. It means catalysis can be triggered via different input energies such as electromagnetic, mechanical, and thermal vibration. The driving factors for the improved performances are associated with the internal electric field due to non-centrosymmetry. In this regard, the piezocatalysis process is one such process where the non-centrosymmetric structure of the ferroelectric material act as a piezocatalyst for water remediation applications. In the piezocatalysis process, mechanical stress/energy causes charge separation resulting in induced polarization in ferroelectric material, which is used in water splitting, organic dyes, and organic compound synthesis applications [21,22,23,24]. BaTiO_3_, BiFeO_3_, Pb(Zr_0.52_Ti_0.48_)O_3,_ LiNbO_3,_ Bi_4_Ti_3_O_12,_ etc. are some of the ferroelectric materials with inbuilt spontaneous polarization that are utilized in water cleaning applications through the piezocatalysis process [25,26,27,28,29]. Recently, researchers have combined piezocatalysis and photocatalysis processes into one system to enhance the transfer and separation of charges, thereby overall improving the photocatalytic performance [30].

Piezoelectric materials, in particular, generates internal electric fields that separate photogenerated electrons and holes with opposing charges in photocatalysis, thereby improving photocatalytic activity. When a catalyst is allowed to work as a piezocatalyst and photocatalyst, by combining photocatalysis and piezocatalysis processes in one system, charge transfer and separation are accelerated, and high photocatalytic performance is achieved [30].

It is noted that ferroelectric materials can only demonstrate piezoelectric nature after electrical poling. Poling is the process of alignment of dipoles in a unidirectional way. It can be stated that the catalytic performances of ferroelectric material (without poling) will not be very effective. Additionally, in the case of ferroelectric ceramic powder, poling with electrodes is not possible. Thus, for efficient conversion to single-domain grains in ferroelectric powder, the corona poling technique can be applied only. The effect of poling was reported on a few ferroelectric ceramics where significant improvement was observed [31].

Due to the versatile properties of BaTiO_3_-based ceramics, its various derivatives (Calcium (Ca), Zirconium (Zr), and Strontium (Sr) as dopants) are studied in piezocatalysis, photocatalysis, and piezo-photocatalysis [17,19,20]. As there are promising results from doping Sr and Zr in BaTiO_3_ solid solution, the multicatalytic properties of BaTiO_3_ ceramic can be improved by simultaneously introducing Sr and Zr elements. Excellent electrical properties have been reported for Sr and Zr in BaTiO_3_ solid solutions [32,33]. Thus, the current study focuses on the photocatalytic, piezocatalytic, and piezo-photocatalytic performance of 0.5Ba(Zr_0.2_Ti_0.8_)O_3_-0.5(Ba_0.7_Sr_0.3_)TiO_3_ composition (BST–BZT). BST-BZT has not been examined for any catalytic processes for water remediation application. The study BST-BZT is reported for various catalytic characteristics with the effect of poling on the same.

## 2. Experimental

### 2.1. Synthesis of BST-BZT Composition

In the present study, BST-BZT composition was synthesized using the conventional solid oxide reaction route. Barium carbonate (BaCO_3_) and Titanium dioxide (TiO_2_) were purchased from Loba Chemie PVT. LTD, Mumbai, Maharashtra, India (purity ~99%), Zirconium Dioxide (ZrO_2_), and Strontium Dioxide (SrCO_3_) was purchased from Fisher Scientific India PVT. LTD, Mumbai, Maharashtra, India (purity ~99%) were mixed according to their stoichiometric ratio using a mortar and pestle. To attain homogeneity, acetone was used as a mixing medium. Furthermore, the mixed powder was subjected to a calcination temperature of 1350 °C for 6 h in an electric furnace (Nabertherm, Germany).

### 2.2. Characterization

The phase formation of the BST-BZT sample was revealed using X-ray diffraction data through a Smart Lab X-ray diffractometer (Rigaku Corporation, Tokyo, Japan) in the range of 20–70° at a rate of 0.03°/s. Additionally, Raman spectroscopy was employed for detecting the vibrational modes present in the BST-BZT samples. For this, a 532 nm wavelength green laser was employed on the sample with a power of 25 watts. A field emission scanning electron microscope (FE-SEM) (FEI SEM NOVA Nanosem 450, Hillsboro, OR, USA) was employed for microstructure and surface morphology analysis. The elements detection in BST-BZT composition was performed using X-ray photoelectron spectroscopy (XPS technique) with an X-ray photoelectron spectrophotometer (Thermo scientific, Model: NEXSA, Waltham, MA, USA) with a micro-focused X-ray (400 µm, 72 Watt, and 12,000 V). A monochromatic Al-Kα source with energy, hν = 1486.6 eV with a hemispherical analyzer and a 128-channel plate detector was used for the XPS analysis. The pass energy for the survey scan was 200 eV, whereas 50 eV was used for a core scan. A UV-visible spectrophotometer (SHIMADZU-2600, Tokyo, Japan) aided in the elucidation of absorbed light in samples and for quantification dye degradation.

### 2.3. Poling of BST-BZT Sample

**Figure 1:**
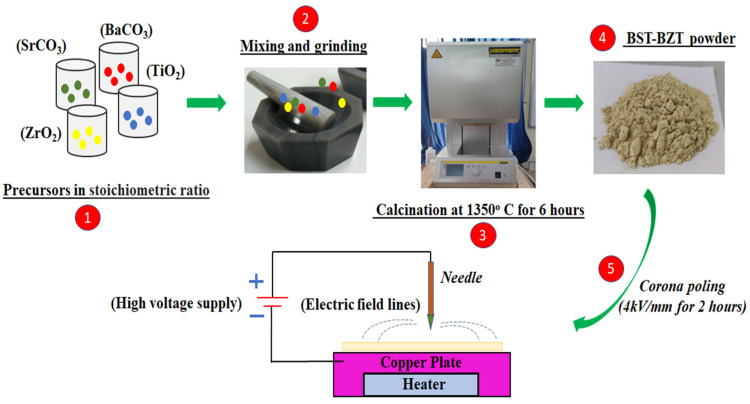
Schematic representation of BST-BZT composition preparation and Corona poling.

The BST-BZT powder was poled through an in-house fabricated Corona poling setup. The powder was placed on the ceramic plate (heated ~40 °C) where it was poled at 4 kV/mm for 2 h. Figure 1 shows the schematic representation of the BST-BZT composition preparation using a solid route reaction method and poling of BST-BZT powder by the corona poling technique.

### 2.4. Piezocatalysis, Photocatalysis, and Piezo-Photocatalysis Experiments

The piezocatalysis, photocatalysis, and piezo-photocatalysis experiments were performed on ~5 mg/L concentrated, 10 mL of organic cationic dye named “Methylene Blue” (MB) dye. For piezocatalysis, a conventional ultrasonicator (150 W and 40 kHz) was used, whereas for the photocatalysis process 2 bulbs (Havells) of 15 watts each were used. For piezo-photocatalysis, the two systems, i.e., piezocatalysis and photocatalysis setups were combined for conducting the experiments. Initially, 0.1 g of the BST-BZT powder was immersed in MB dye overnight for adsorption powered by a magnetic stirrer. Then, the adsorbed powder was subjected to visible light, vibrations, and visible light plus vibrations to assess photocatalysis, piezocatalysis, and piezo-photocatalysis process for MB dye degradation. In each experiment after every 30 min, 1 mL of dye was taken out, centrifused and its absorbance was checked using a UV-visible spectrophotometer (SHIMADZU-2600, Tokyo, Japan) for dye degradation quantification.

## 3. Results and Discussion

**Figure 2:**
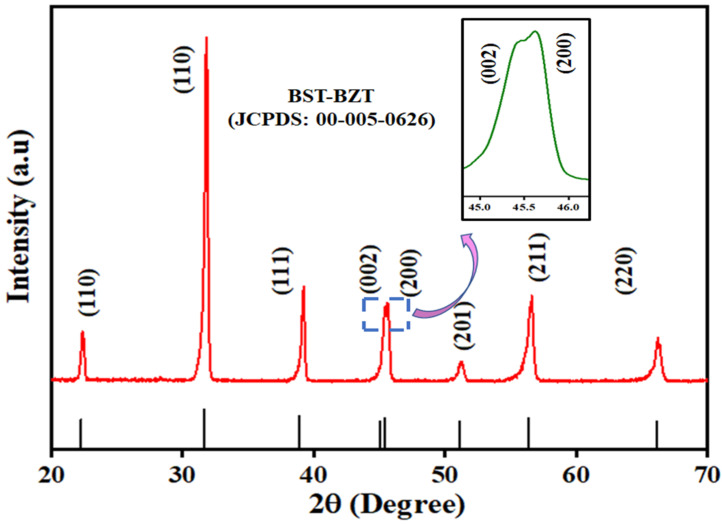
XRD pattern of BST-BZT ceramic powder.

**Figure 3:**
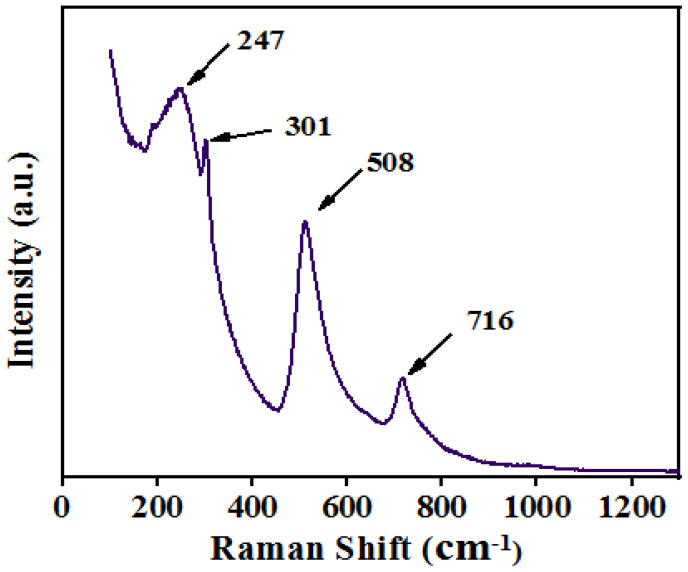
Raman spectrum of BST-BZT powder.

Figure 2 shows the XRD pattern for the BST-BZT composition in a range of 20–70°. The obtained peaks were in accordance with tetragonal BaTiO_3_ ceramics (JCPDS No: 00-005-0626) thus confirming that Zirconium (Zr) and Strontium (Sr) were well doped in BaTiO_3_ ceramics and no presence of an impurity in the synthesized BST-BZT powder [8]. There is a splitting of the peak at a ~45° angle into two where the peaks corresponding to (002) and (200) Bragg planes as shown in the magnified view of Figure 2, where the intensity of the peak corresponding to the (200) plane was more compared to the (002) plane, which indicates that there is a maximum possibility that BST-BZT crystallized in tetragonal phase. Further information about structural formation in BST-BZT composition was revealed through Raman spectroscopy. Figure 3 shows the Raman spectrum between 150–1250 cm^−1^ wavenumber.

**Figure 4:**
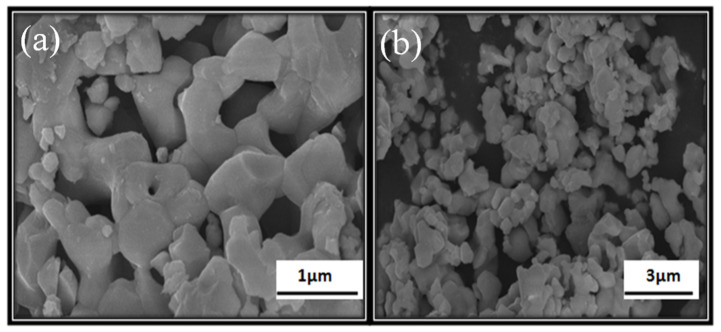
SEM images of BST-BZT at certain magnification at scale of (**a**) 1 µm, and (**b**) 3 µm.

Dominating peaks were observed at ~247, ~304, ~508, and ~716 cm^−1^ wavenumbers. A dip at ~180 wavenumber (cm^−1^) is observed, which is due to the A1(TO2) anti-symmetry mode. Furthermore, the peaks at ~255 cm^−1^ and ~508 cm^−1^ are due to A1 symmetry caused by transverse optical modes, whereas the peak at ~301 cm^−1^ is due to B1 mode and ~716 cm^−1^ is a consequence of A1(LO3)E(LO) mode, which may indicate the tetragonal phase is formed in a BST-BZT composition [34,35]. Through the XRD and Raman spectroscopy results, there is a high possibility that BST-BZT crystallized in the tetragonal phase. However, there are some works reported in the literature where with an increase in the concentration of dopant in BaTiO_3_ ceramic, the BaTiO_3_ crystallized in pseudo tetragonal/cubic phase [36,37]. Figure 4a,b shows the surface morphology of the synthesized BST-BZT composition at a 1 and 3 µm scale obtained from scanning the electron microscope at a certain magnification. The morphology of the BST-BZT composition shows an irregular shape of particles with smooth edges.

**Figure 5:**
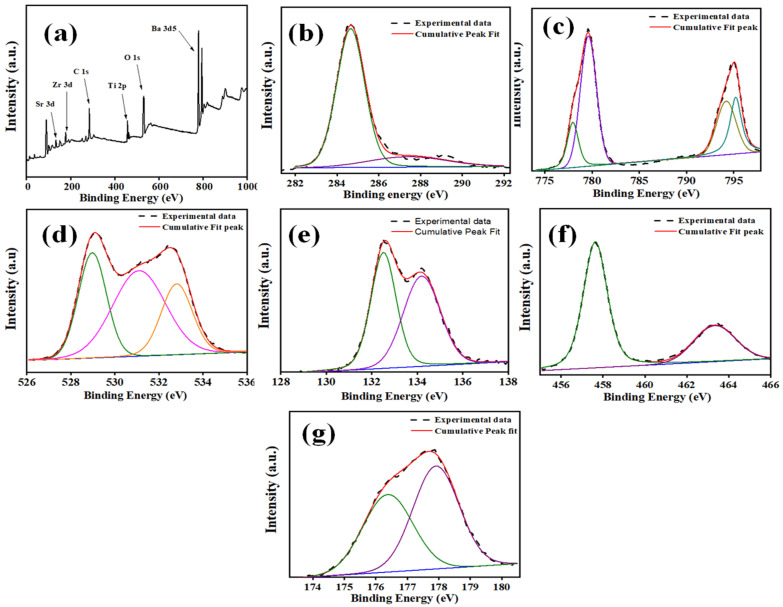
(**a**) XPS survey showing elemental composition in BST-BZT, (**b**) C1s spectrum, (**c**) Ba 3d spectrum, (**d**) O1s spectrum, (**e**) Sr3d spectrum, (**f**) Ti2p spectrum, (**g**) Zr 3d spectrum.

**Figure 6:**
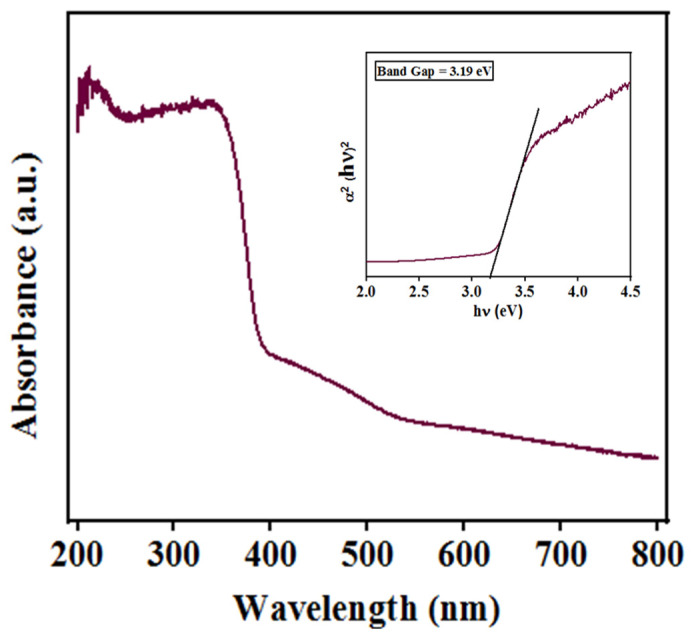
Absorbance vs. wavelength plot showing band gap (Tauc’s plot inset).

The chemical composition of the BST-BZT powder was determined using the XPS technique. Figure 5a shows the XPS survey (0–1000 eV binding energy) of BST-BZT ceramic powder revealing that Ba, Zr, Sr, Ti, and O elements were present in the composition. Furthermore, the XPS spectrum was calibrated according to the binding energy of carbon (~285 eV). Each element was deconvoluted into the peaks using linear as a background. In the C1s spectrum shown in Figure 5b, the obtained binding energy at ~284.6 eV and ~288 eV is due to the C-C/C-H bond and carbonates [38]. The Ba XPS survey in Figure 5c shows that Ba3d3/2 and Ba3d5/2 peaks can be fitted around at a difference of ~1.5 eV. The binding energies ~794.16 and ~778 eV are due to the Ba atoms in BST-BZT ceramics, whereas the peaks around ~779.6 and ~795.18 eV are assigned to Ba atoms obtained during decomposed barium carbonate layers [39]. The O1s spectra can be deconvoluted into three peaks shown in Figure 5d centered at ~528.97 eV, ~531.09 eV, and ~532.83 eV, which are assigned to the oxygen lattice in the sample, oxygen vacancy, and adsorbed oxygen, respectively [40]. Although a peak at ~531.09 eV does not directly indicate the detection of oxygen vacancies, it helps in identifying the adsorbed O^−^, O_2_^−^, and –OH groups trapped in formal oxygen vacancies in synthesized material [41,42], whereas higher binding energy at ~532.83 eV is generally due to the chemisorbed and oxygen-dissociated species. Furthermore, the Sr3d peaks can be deconvoluted at ~132.5 and 134.32 eV binding energies as shown in Figure 5e. The peaks shown in Figure 5f fitted perfectly at binding energy ~458 eV and ~464 eV, which is attributed to the Ti2p3/2 and Ti2p1/2 core level with Ti^4+^ cations [43]. Figure 5g shows profiles representing two-spin orbit peaks of Zr, i.e., the 3d5/2 peak at a binding energy of ~176.4 eV and the 3d3/2 peak at a binding energy of ~178.0 eV. Figure 6 shows the absorbance spectrum of BST-BZT powder sample in a range of 200–800 nm wavelength. The powder sample absorbs much less energy in the range of 500–800 nm wavelength, however there is a gradual increment in the absorbance in the range of 415–550 nm wavelength. After the 415 nm wavelength, there is a sudden rise in the absorption revealing the maximum absorption of the light up to a 200 nm wavelength. Furthermore, the energy band gap of the synthesized BST-BZT composition was calculated through Tauc’s relation using Equation (1) [44,45].

(1) $$\alpha \left(h\nu \right)=B{\left(h\nu -Eg\right)}^{m}$$

where, “B” is the energy independent coefficient, “α” is the coefficient of the absorption, “Eg” is the energy band gap of the synthesized sample, “h” is the plank’s constant, “*ν*” is the frequency of the light, and “m” represents the nature of the electronic transition responsible for optical absorption. The energy band gaps (direct and indirect) are determined by using “m” as 1/2 and 2, respectively. The energy band gap of BST-BZT composition was found to be ~3.19 eV using a Tauc’s plot as shown in Figure 6. There is not much considerable change in the band gap of the BST-BZT composition compared to the ~3.2 eV band gap of BaTiO_3_ (tetragonal phase) [8].

Under individual and coupled catalytic conditions, the contribution of photocatalytic and piezoelectric properties to degradation was assessed as follows: photocatalysis, piezocatalysis, and piezo-photocatalysis through both unpoled and unpoled BST-BZT powder. In the present study, methylene blue (MB) dye has been selected as an indicative organic pollutant in water. For the assessment of MB dye degradation through photocatalysis, piezocatalysis, and piezo-photocatalysis processes, initially, 0.1 g of BST-BZT powder was immersed in 10 mL of ~5mg/L concentrated MB dye overnight. This was performed to attain an adsorption–desorption equilibrium between the catalyst (BST-BZT composition) and MB dye. Once the adsorption–desorption equilibrium was attained, the BST-BZT powder was subjected to a visible light source, vibrations, and visible light plus vibrations to quantify the weakening of the MB dye. The dye-containing BST-BZT composition was stirred using a magnetic stirrer while placing it in a dark environment for distinguishing adsorption and catalytic processes.

**Figure 7:**
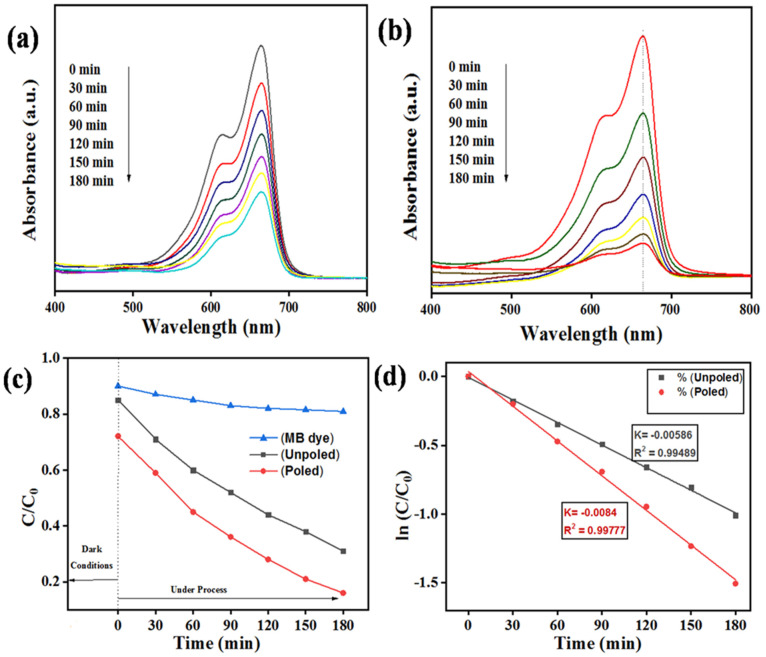
(**a**) Absorbance spectra of MB dye through photocatalysis process under visible light using 0.1 g of unpoled BST-BZT composition, (**b**) absorbance spectra of MB dye through photocatalysis process under visible light using 0.1 g of poled BST-BZT composition, (**c**)% degradation of MB dye after 3 h using 0.1 g of unpoled and poled BST-BZT powder through photocatalysis process, and (**d**) kinetic parameter of MB dye using 0.1 g of unpoled and poled BST-BZT powder under visible light.

Figure 7a,b show the absorbance spectra of MB dye taken at an interval of 30 min and continuing up to 3 h using 0.1 g of both unpoled and poled BST-BZT samples through the photocatalysis process.

The degradation of MB dye was measured by following Equation (2) [46,47].

(2) $$\mathrm{Dye}\;\mathrm{degradation}\;(\%)=\left(1-\frac{C}{C_{o}}\right)\times 100=\left(1-\frac{A}{A_{o}}\right)\times 100$$

where, “A” and “$A_{o}”$ are the absorbance peak, and “C” and $”C_{o}”$ denote the concentration of MB dye at time = “t” mins and at a time “t” = 0 min. The degradation of the MB dye was checked using a UV-Visible spectrophotometer by noting the absorbance peak of the MB dye, which occurs at ~664 nm wavelength [48]. Clearly, there is a decrement in the absorbance spectrum after every 30 min evidencing the weakening of the MB dye. Furthermore, Figure 7c shows the percentage of MB dye degradation achieved after every 30 min using both unpoled and poled BST-BZT samples. The degradation of MB dye was ~10% in 3 h without using any catalyst (control sample) as shown in Figure 7c, whereas Figure 7d shows the kinetic rate constant of MB dye degradation through the photocatalysis process, which is calculated by following Equation (3) [49,50].

(3) $$\ln\left(\frac{C}{Co}\right)=-kt$$

**Figure 8:**
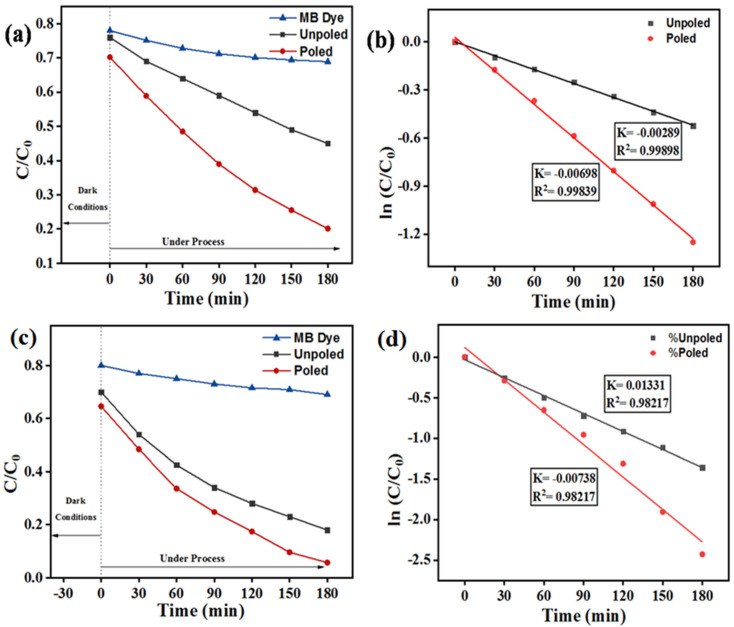
(**a**) % degradation of MB dye after 3 h using 0.1 g of unpoled and poled BST-BZT powder through piezocatalysis process, (**b**) kinetic parameter of MB dye using 0.1 g of unpoled and poled BST-BZT powder in piezocatalysis process, (**c**) % degradation of MB dye after 3 h using 0.1 g of unpoled and poled BST-BZT powder through piezo-photocatalysis process, and (**d**) kinetic parameter of MB dye using 0.1 g of unpoled and poled BST-BZT powder in piezo-photocatalysis process.

Using unpoled and poled BST-BZT composition, the kinetic rate “k” was found to be 0.00586 and 0.0084 min^−1^, respectively, for the photocatalysis process. Figure 8a,c show the percentage of MB dye degradation using an unpoled and poled BST-BZT sample under piezocatalysis and piezo-photocatalysis process, whereas Figure 8b,d show the kinetic rate for MB dye weakening by using an unpoled and poled BST-BZT sample under a piezocatalysis and piezo-photocatalysis process, respectively. The kinetic rate under the piezocatalysis process was found to be 0.00289 and 0.00698 min^−1^ using an unpoled and poled BST-BZT sample, whereas 0.00738 and 0.01331 min^−1^ was a kinetic rate using unpoled and poled BST-BZT sample under piezo-photocatalysis process, respectively.

**Figure 9:**
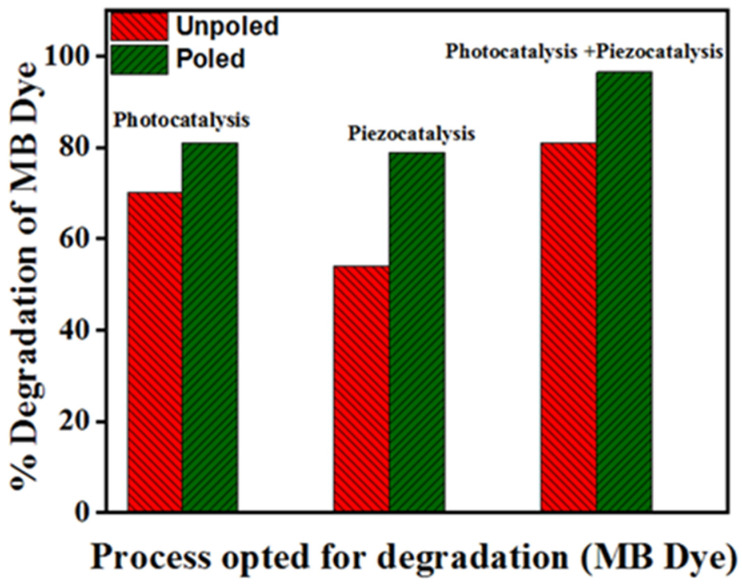
% Degradation achieved by using unpoled and poled BST-BZT sample in photocatalysis, piezocatalysis, and piezo-photocatalysis process.

There is an effect of poling in the BST-BZT sample for MB dye degradation. The degradation achieved by unpoled and poled BST-BZT samples were ~70 and ~81 through photocatalysis, ~54 and ~79 through piezocatalysis, and 81 and 97% through piezo-photocatalysis processes, respectively. Figure 9 shows a comparison of unpoled and poled BST-BZT samples aiding in MB dye degradation through piezocatalysis, photocatalysis, and piezo-photocatalysis. Coupling piezocatalysis and photocatalysis effect resulted in ~81 and 97% degradation of MB dye from unpoled and poled BST-BZT samples in 3 h. Based upon the first order pseudo kinetics, the kinetic rate of the piezo-photocatalysis process is 1.3 and 1.6 times more compared to the photocatalysis process, while, compared to piezocatalysis, 2.5 and 1.9 times more kinetic rate was achieved through an unpoled and poled BST-BZT sample, respectively.

When the light of a specific wavelength is exposed to a photocatalyst, the electron (e^−^) in the valence band (VB) gains enough energy to leave the valence band and enter the conduction band (CB), leaving an equal number of holes (h^+^) in the valence band. This formed electron-hole pair reacts with water components in the dye solution, forming reactive species that attack the dye molecules. For this, the conduction band potential should be more negative than the potential of O_2_/·O_2_ by which the electron (e^−^) can reduce to ·O_2_^−^ from O_2_, whereas the valance band potential should be higher than the potential of H_2_O/·OH, thus ·OH can be generated thermodynamically and oxidation may occur, resulting in the weakening of the organic dye [51]. The calculated energy band gap of the BST-BZT composition, i.e., 3.19 eV, should be limited to the ultra-violet region of electromagnetic waves (100–400 nm wavelength). However, in the present study, in the photocatalysis process, significant degradation was achieved from both poled and unpoled BST-BZT samples. The photoactivity could be due to a gradual increment in absorption in the range of 415–550 nm wavelength as shown in Figure 6. The previous literature also showed such results where the energy band gap is greater than 3.0 eV and showed photoactivity in the visible region [52].

However, in the piezocatalysis process, shock waves exert pressure on the catalyst, resulting in a direct piezoelectric effect in the synthesized samples contained within the dye. The charge gets separated, i.e., positive charge (holes) and negative charge (free electron) onto the surface of the BST-BZT sample, which participates in the redox reaction. At the same time, this process would result in a novel property of piezocatalysts, as the piezoelectric potential tilts both CB and VB of the BST-BZT sample. Additionally, in the piezocatalysis process, shock waves create cavities forming bubbles, which grow and ultimately burst during ultrasonication. The formation/growth/collapse of the bubbles lead to an increase in the localized temperature up to 4000–5000 K and generate shock waves up to ~10^8^ Pa pressure [53]. This phenomenon is called thermolysis or sonolysis due to which the degradation of the dyes may take place. However, to decrease the thermolysis/sonolysis effect, ice-cold water (<15 °C) was used as a vibration medium. Figure 8a,c show the degradation of the control sample (without using the BST-BZT sample). Sonocatalysis cannot be avoided under ultrasonication waves. However, the impact of poling supports the intrinsic piezocatalysis processes.

**Figure 10:**
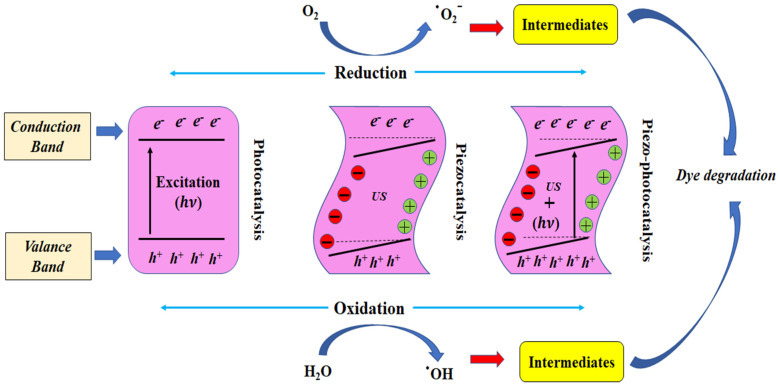
A plausible mechanism of photocatalysis, piezocatalysis, and piezo-photocatalysis process.

The photogenerated electrons and holes in the photocatalysis process could recombine, resulting in a decrease in charge carriers and thus can obstruct the degradation performance. Furthermore, the piezoelectric effect would separate charges on the surface of the BST-BZT sample when mechanical force was applied. As the piezoelectric potential tilts both CB and VB of the BST-BZT sample, the tilted VB would effectively attract the h^+^ to oxidize H_2_O to form OH, while the tilted CB would accumulate e^-^ to reduce O_2_ to generate O_2_ because the tilted CB and VB after incorporating the piezoelectric effect into photocatalysis photogenerated electrons and holes are more easily excited to participate in the redox reactions for pollutant degradation [54]. The induced piezoelectric field has improved the separation efficiency of photogenerated electron-hole pairs. The induced piezoelectric field separates more photogenerated electrons and holes, which then migrate to the opposite surface of the catalyst and participate in redox reactions, resulting in a significant increase in catalytic degradation activity and as a result enhance catalytic activity in the piezo-photocatalysis process compared to piezocatalysis and photocatalysis processes. Figure 10 shows the plausible mechanism of photocatalysis, piezocatalysis, and piezocatalysis processes separately.

In contrast to piezoelectric sensing, actuating, and energy harvesting applications, piezocatalytic can occur in unpoled samples. This is because the piezocatalysis process necessitates the local interaction of pollutant molecules with the surfaces of piezoelectric materials. This enables multiple domains (as individual piezoelectric) in unpoled ferroelectric material particles to participate in catalytic reactions. However, polling, on the other hand, can significantly improve performance by creating a high surface electric potential on the surfaces by aligning the dipoles in a specific direction [31]. This is supported by ~54% and ~79% degradation of MB dye achieved by unpoled and poled BST-BZT samples, respectively, in 3 h of ultrasonication. Additionally, poled ferroelectric ceramics have shown enhanced photocatalytic activities. This is due to the reason that poling accelerates the separation and migration of electron-hole pairs and results in increased photocatalytic activity [55,56]. Additionally, in the case of the piezo-photocatalysis process, enhanced MB dye degradation was achieved using poled BST-BZT compared to an unpoled BST-BZT sample.

**Figure 11:**
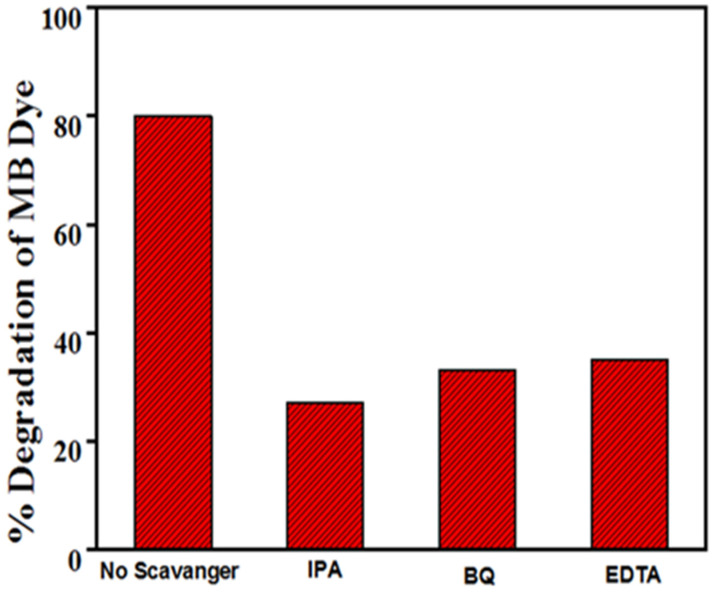
% Degradation achieved by using EDTA, IPA, and BQ scavengers in piezocatalysis process using poled BST-BZT sample in 3 h.

The photocatalysis, piezocatalysis, and piezo-photocatalysis processes generate attacking species such as holes (h^+^), hydroxyl radicals (OH), electrons (e^-^), and superoxide radicals (O_2_), which are responsible for the degradation of the MB dye. However, only one of the attacking species has dominant characteristics that cause dye degradation. A specific scavenger traps specific attacking species, which means that if that specific attacking species is trapped, dye degradation will not occur to the extent that it would have without that specific scavenger. The scavenger test was used to identify the reactive species responsible for MB dye degradation using poled the BST-BZT sample. In the current study, a scavenger test was performed in a piezocatalysis process by adding 1000 µL of Ethylene diamine tetra acetic acid (EDTA), Isopropanol (IPA), and Benzoquinone (BQ) scavengers to 10 mL of 5 mg/L concentrated MB dye. Figure 11 depicts the degradation of MB dye by scavengers IPA, BQ, and EDTA at 26%, 36, and 38%, respectively. Scavengers such as EDTA, IPA, and BQ capture reactive species such as OH, h^+^, and O_2_ [17]_._ Figure 11 shows that among all scavengers, MB dye containing IPA has shown the least degradation, indicating the least participation of OH radical in 3 h of ultrasonication. This indicates that scavenger IPA has trapped the OH radical, which was the primary attacking species in the piezocatalysis process.

## 4. Conclusions

The ferroelectric ceramic 0.5Ba(Zr_0.2_Ti_0.8_)O_3_-0.5(Ba_0.7_Sr_0.3_)TiO_3_ composition (BST–BZT) was synthesized via the solid-state reaction route. The synthesized BST-BZT ceramic was studied for multicatalytic activity, i.e., photocatalysis, piezocatalysis, and piezo-photocatalysis for degrading MB dye. The degradation was found to be ~70, ~54 and ~81% for photocatalysis, piezocatalysis, and piezo-photocatalysis processes, respectively. Clearly, the performance was improved by the synergistic effect of photocatalysis and the piezocatalysis process. Further in the study, the BST-BZT sample was poled using the corona poling technique where significant enhancement was observed compared to the unpoled BST-BZT powder sample. The kinetic rate was 1.4, 2.4, and 1.9 times more when poled the BST-BZT sample was used compared to the unpoled BST-BZT sample in photocatalysis, piezocatalysis, and piezo-photocatalysis processes, respectively. Thus, the present study brings out a novel study by combining photocatalysis and piezocatalysis processes for organic pollutant removal, which can be further accelerated by the electric poling of ferroelectric catalysts.

## Data Availability

The data that support the findings of this study are available from the corresponding author upon reasonable request.
